# Chemical Profiling and Geographic Differentiation of Ugandan Propolis by GC-MS Through Chemometric Modelling

**DOI:** 10.3390/molecules30224435

**Published:** 2025-11-16

**Authors:** Ivan Kahwa, Leonard Kaysser, Rapheal Wangalwa, Susan Billig, Jonans Tusiimire, Claudia Wiesner

**Affiliations:** 1 Institute for Drug Discovery, Department of Pharmaceutical Biology, Faculty of Medicine, Leipzig University, 04317 Leipzig, Germany; leonard.kaysser@uni-leipzig.de; 2 Department of Pharmacy, Faculty of Medicine, Mbarara University of Science and Technology, Mbarara P.O. Box 1410, Uganda; jonanstusiimire@must.ac.ug; 3 Pharm-Biotechnology and Traditional Medicine Centre, Mbarara University of Science and Technology, Mbarara P.O. Box 1410, Uganda; wangarapha@must.ac.ug; 4 Institute of Analytical Chemistry, Faculty of Chemistry, Leipzig University, 04103 Leipzig, Germany; billig@uni-leipzig.de; 5 Department of Biology, Faculty of Science, Mbarara University of Science and Technology, Mbarara P.O. Box 1410, Uganda; 6 German Center for Integrative Biodiversity Research (iDiv) Halle-Leipzig-Jena, 04103 Leipzig, Germany

**Keywords:** Ugandan propolis, GC-MS analysis, agroecological variation, chemometric profiling, terpenoid biomarkers

## Abstract

Propolis is a resinous substance collected by honeybees, and its long-known bioactivity urged research on its exact composition on active ingredients. It was suggested that chemical composition reflects the botanical sources and environmental conditions of its origin; however, information on differences related to geographical origin is still incomplete. Therefore, this study aimed to characterise the volatile and semi-volatile chemical constituents of Ugandan propolis from nine agro-ecological zones using headspace gas chromatography–mass spectrometry (HS-GC-MS) and derivatisation-based GC-MS, coupled with multivariate statistical analysis. In total, 213 volatile and 169 non-volatile compounds were tentatively identified, including monoterpenes (α-pinene), sesquiterpenes (α-copaene), triterpenoids (β-amyrin acetate), diterpene resin acids (abietic acid), phenolic acids (caffeic acid), alkylresorcinols (bilobol) and many others. Multivariate chemometric modelling using partial least-squares discriminant analysis (PLS-DA), orthogonal PLS-DA (oPLS-DA) showed strong geographic discrimination of samples (Q^2^ > 0.90) for several district comparisons. Heatmap clustering and variable importance in projection (VIP) analysis identified chemical markers. Notably, oPLS-DA revealed excellent discrimination between Nakasongola and Bushenyi, and between Adjumani and Bushenyi, in both volatile and non-volatile datasets. The findings provide the first comprehensive chemical profiling of Ugandan propolis, demonstrating the utility of combined GC-MS approaches and multivariate analysis for regional differentiation. This work lays the groundwork for standardising propolis preparations and establishing appropriate quality control in pharmacological applications.

## 1. Introduction

Propolis, also known as “bee glue”, is a complex resinous mixture created by honeybees using saliva, plant exudates, wax, and pollen [1]. It serves to seal small openings, cracks and crevices, while also inhibiting the development of bacteria, fungi and other pathological microorganisms [2]. Traditionally, different communities have used it to treat respiratory conditions, dental issues, wounds and burns, low immunity, skin problems, and digestive disorders [3,4]. It has garnered significant scientific and medical attention for its diverse pharmacological properties, including antimicrobial, antioxidant, antifungal, anticancer, and anti-inflammatory effects, among others [5,6,7]. The therapeutic benefits of propolis are largely attributed to its rich content of bioactive compounds, especially polyphenols (such as flavonoids and phenolic acids) and terpenoids, which synergistically inhibit microbial growth and neutralise oxidative stress [5]. For instance, flavanones such as pinocembrin and galangin, and caffeic acid derivatives (phenolic acids), contribute to the antibacterial and antifungal properties of propolis, while prenylated phenolics and triterpenes provide additional anti-inflammatory and antioxidant activity [8,9,10,11].

Raw propolis usually consists of about 50 to 70% plant resins and balsams, 30% waxes and fatty acids, 10% essential oils, and the remaining components being pollen and other organics [12,13]. More than 300 to 500 unique compounds have been identified in numerous types of propolis from locations around the world; these include flavonoids, phenolic acids, terpenoids, fatty acids, and aromatic aldehydes or alcohols [14,15]. For example, the resin and pollen balm of Okinawan propolis were shown to be rich in flavonoids such as chrysin, galangin, pinocembrin, pinobanksin acetate, prenylflavonoid, isonymphaeol-B, and nymphaeol-A, -B and -C [16]. Propolis also contains phenolic acids (cinnamic acid, coumaric acid, caffeic acid, ferulic acid, isoferulic acid) and their esters, as well as polysaccharides [17,18]. Various propolis samples exhibit a range of terpenoids, including monoterpenes such as terpinen-4-ol and limonene, sesquiterpenes like α-copaene and β-elemene, diterpenes such as dehydroabietic acid and abietic acid, triterpenes including β-amyrin acetate and lupeol acetate, as well as phytosterols like fucosterol and stigmasterol acetate [19,20,21,22].

Importantly, the chemical composition of propolis is highly variable depending on plant source (s), season, climate, and geographical location [23]. Since bees gather various resins from local plants, propolis from different regions often shows significant variation in chemical composition [24,25]. For instance, propolis collected from temperate zones, which is mainly acquired from poplar trees and other plant species such as birch, beech, alder, spruce, poplar, horse chestnut and elm trees, is high in flavonoid aglycones and phenolic esters [26], but tropical or subtropical propolis (from Africa and South America) may contain higher levels of terpenoids and other unique compounds [27,28,29,30]. In China, the main producer of propolis in Asia, it is primarily derived from *Populus* spp. [31]. The chemical constituents of Chinese propolis are compared to those found in temperate and subtropical regions, despite the country’s varied temperate climates [31,32,33,34]. Despite this global diversity, African propolis remains underrepresented in the scientific literature, especially given its importance across various fields, including health applications [35]. This is especially true for Uganda, where agroecological zones ranging from dry savannahs to highland forests, offer a unique opportunity to explore how flora influences propolis composition [36,37,38].

To analyse this high compound diversity, gas chromatography–mass spectrometry (GC–MS), including headspace (HS-GC–MS) and GC–MS after derivatisation, has been recognised a powerful tool. These analytical techniques can unravel the chemical profile of propolis, especially its volatile and semi-volatile, low molecular weight compounds. For example, using headspace injections (for volatile compounds), Baptista Pereira et al. [39] reported 60 components in propolis, and 76 constituents in its essential oil with GC-MS [40]. Headspace GC–MS (HS-GC–MS) is particularly suitable for analysing volatile organic compounds, i.e., constituents of essential oils and other small compounds [41]. GC–MS of derivatised propolis extracts, on the other hand, can identify less volatile polar metabolites (e.g., sugars, organic acids, polar phenolic compounds, and triterpenes), with chemical derivatisation improving their GC accessibility [42]. Both techniques (HS-GC–MS and GC–MS of derivatised samples) can be combined to create a more comprehensive chemical fingerprint of propolis, encompassing a range of compound volatilities and polarities.

When combined with chemometric methods such as partial least-squares discriminant analysis, these analytical approaches successfully distinguished propolis samples by their geographic origin [43]. In this study, HS-GC–MS and GC–MS analyses following trimethylsilyl derivatisation, were conducted on propolis samples from nine agroecological regions in Uganda. The goal was to develop a detailed chemical profile, examine compositional differences, and identify chemical markers across these zones. This research enhances our understanding of the chemical diversity of Ugandan propolis by examining how plant sources and environmental factors influence its composition, which bioactive ingredients are present, and potential uses.

## 2. Results and Discussion

### 2.1. Propolis Colour Variation Across Sample Sites Representing Agroecological Zones

**Figure 1:**
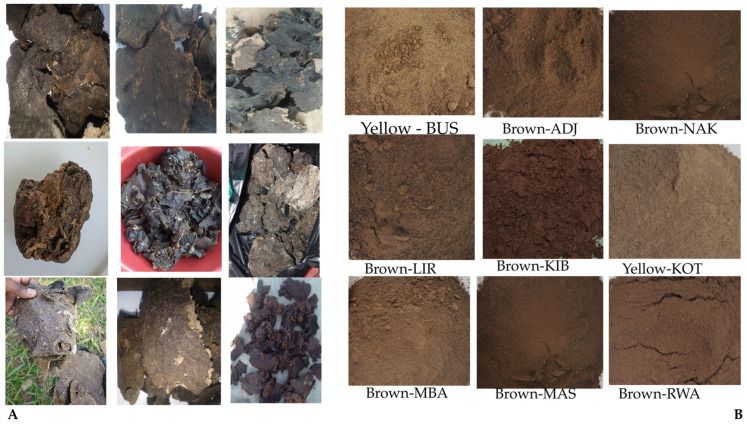
Colour variation in Ugandan propolis from districts representing different agroecological zones. (**A**) Raw propolis showing natural morphological diversity. (**B**) Homogenised samples illustrating district-dependent colour profiles linked to botanical resin sources.

Propolis samples used in this study were collected from districts which represent specific agroecological zones, each with unique climatic conditions (e.g., unimodal and bimodal rainfall of 500–1200 mm/year and temperature of 15–35 °C), soil types (such as ferralsols, acrisols, nitisols, vertisols and luvisols) and plant species and shrubs from moist green forests, savannah grasslands, wood lands and wetlands, all contributing to the chemical composition of propolis produced in these regions [38,44,45]. The selected districts included Masindi (MAS), Lira (LIR), Adjumani (ADJ), Kibuku (KIB), Nakasongola (NAK), Mbarara (MBA), Bushenyi (BUS), Rwampara (RWA) and Kotido (KOT). The visual assessment of these samples revealed notable colour variations. Brown was the most prevalent colour in MAS, LIR, ADJ, KIB, NAK, MBA, and RWA samples. Yellow propolis was identified in KOT and BUS samples, as shown in Figure 1.

The observed colour variations in Ugandan propolis samples are expected to reflect the influence of local flora, resin sources, and ecological conditions. Indeed, the colour of propolis provides an important phenotypic cue to its chemical composition, which is determined by the botanical resin sources exploited by honeybees [28], for instance, woody and resinous plant sources such as pine trees are known to contribute to darker pigmentation and a more viscous texture in propolis [46]. The colours observed here were reported before; similar colours have been documented in other African propolis samples, for example, dark brown propolis from Cameroon and brown propolis from Nigeria [47,48]. Yellow propolis has not yet been reported in most regions of Africa; however, it has been extensively studied in countries like Brazil, Cuba, Turkey, and some parts of Europe, where its constituents have demonstrated various pharmacological properties, including anti-tumour, antiviral, antioxidant, and cytotoxic activities [49,50,51,52].

### 2.2. Chromatographic Profiles of Ugandan Propolis from Different Agroecological Zones

#### 2.2.1. Representative Chromatograms from Propolis Samples Analysed by HS-GC-MS

**Figure 2:**
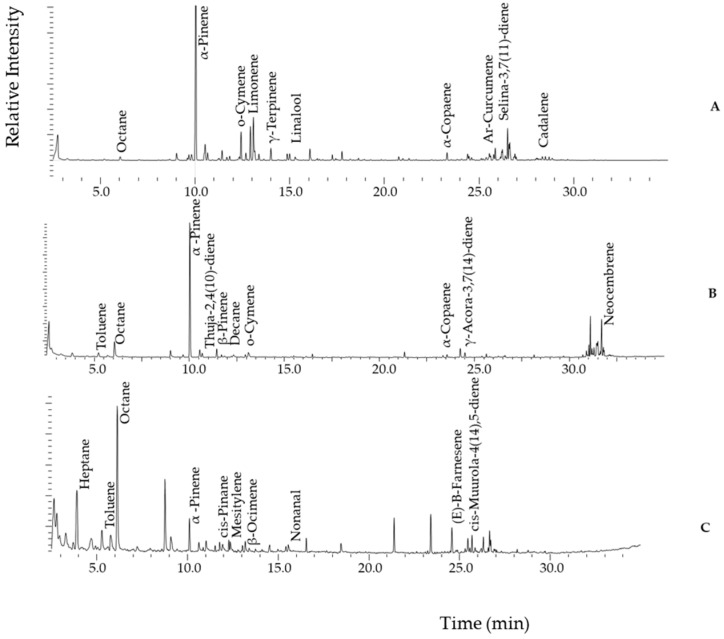
Representative HS-GC-MS chromatograms of selected propolis samples, peaks labelled according to their abundance. (**A**): Bushenyi propolis; (**B**): Mbarara propolis, and (**C**): Kotido propolis.

Headspace gas chromatography–mass spectrometry (HS-GC-MS) analysis of Ugandan propolis revealed various volatile profiles across different districts. Samples from Bushenyi, Mbarara, and Kotido displayed the most chemically diverse and distinct chromatograms (Figure 2), reflecting the broad spectrum of volatiles that characterise Ugandan propolis. The representative chromatograms from Bushenyi (Figure 2A) and Kotido (Figure 2C) samples were dominated by a rich array of monoterpenes and sesquiterpenes, indicative of the botanical sources available to bees in these regions [53]. By contrast, Mbarara (Figure 2B) showed a similarly complex fingerprint but with an additional diterpene signal, suggesting access to taxa rich in higher terpenoids.

For the following detailed discussion of the chemical composition of our propolis samples, tentative identifications were achieved by spectral comparison with the NIST14 mass spectral library, considering the expected elution order and the retention times of substances with rather similar spectra. According to this, monoterpenes included α-pinene, limonene, γ-terpinene, β-ocimene, and nonanal, among others. The sesquiterpene profile was dominated mainly by α-copaene, ar-curcumene, E-β-farnesene, selina-3,7(11)-diene and γ-acora-3,7(14)-diene. A distinctive feature of Mbarara propolis was the exclusive presence of diterpenes, i.e., neocembrene and 13-epi-manoyl oxide. Diterpenes are well recognised but less widely distributed as constituents of propolis; their occurrence is tightly coupled to specific botanical sources and often tracks tropical or resin-rich floras. From a botanical perspective, cembrene-type and labdane/manoyl-oxide–type diterpenes are associated with resinous plants such as coniferous trees (pine trees), which may be more accessible in the Southern Rangelands [54].

#### 2.2.2. Representative GC-MS Chromatograms from Derivatised Propolis Samples

**Figure 3:**
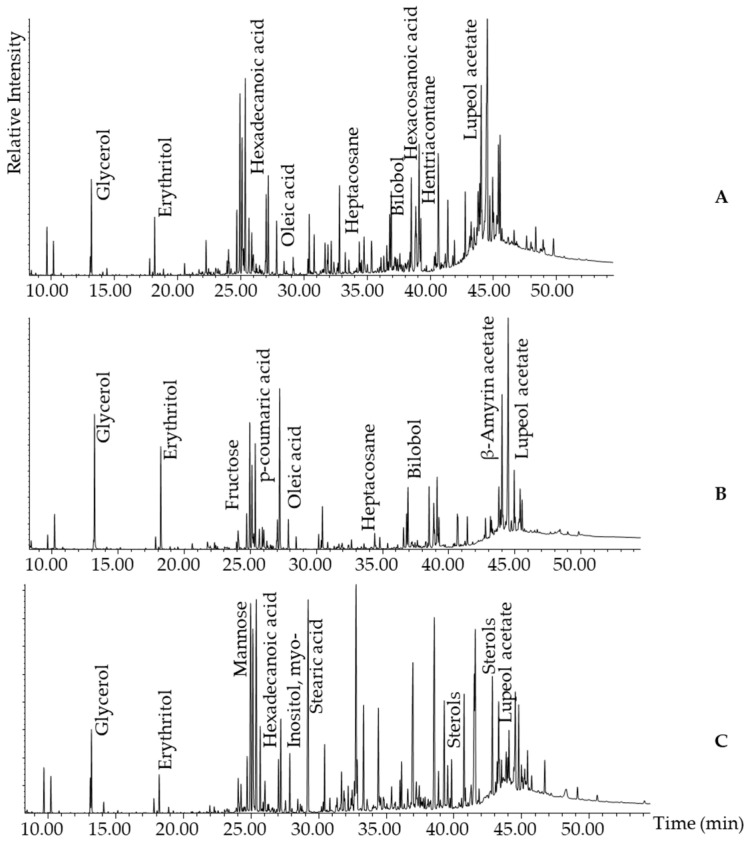
Representative GC-MS chromatograms for non-volatiles with peaks labelled according to their abundance. (**A**): Bushenyi propolis; (**B**): Rwampara propolis, and (**C**): Adjumani propolis.

To enhance the volatility, detectability, and stability of semi-volatile components in propolis for the GC-MS process, we derivatised all samples using trimethylsilylation. The representative chromatograms from the derivatised propolis dataset, including samples from Bushenyi, Rwampara, and Adjumani (see Figure 3), showed chemical complexity and distinct patterns.

All three samples had many, well-resolved peaks from approximately 10 to 46 min, indicating a diverse range of non-volatile compounds. A similar profile was observed within a 10–30 min retention time window, consisting mainly of sugars (e.g., mannose, fructose) and their derivatives (i.e., sugar acids, sugar alcohols), fatty acids (e.g., oleic acid, hexadecanoic acid, and stearic acid), and phenolic acids (e.g., *p*-coumaric acid). Furthermore, from 30 min onward, all samples shared the characteristic presence of the triterpenoid lupeol acetate. However, in Bushenyi (Figure 3A) and Rwampara (Figure 3B), a resorcinol, bilobol, was observed instead, along with long-chain aliphatic hydrocarbons such as heptacosane. In contrast, the Adjumani sample (Figure 3C) was dominated by intense, late-eluting peaks primarily composed of sterols. This suggests a higher abundance of non-polar or high-molecular-weight compounds in Adjumani, which are either absent or significantly less intense in the Bushenyi and Rwampara samples.

### 2.3. Detailed Volatile and Semi-Volatile Chemical Composition of Ugandan Propolis

**Figure 4:**
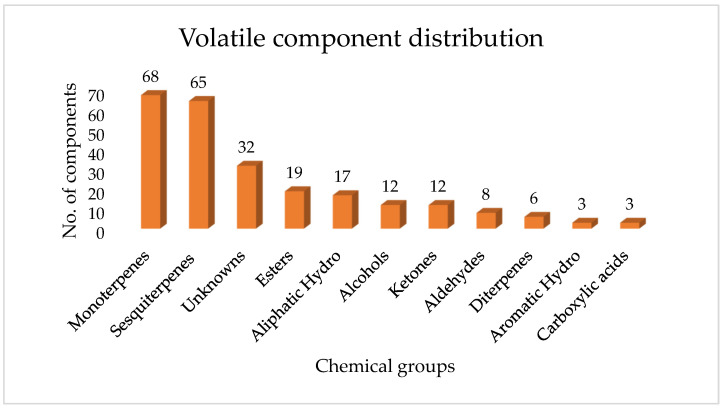
Distribution of the volatile composition per chemical group of Uganda propolis grouped in different phytochemical classes.

The detected volatile compounds were sorted by median peak area (Table S1 in Supplementary File S1) and tentatively classified into major chemical categories, with monoterpenes contributing the most, followed by sesquiterpenes. Other classes included diterpenes, esters, alcohols, ketones, aldehydes, carboxylic acids, aliphatic and aromatic hydrocarbons, and many unknown compounds, as shown in Figure 4.

According to the median peak area of the selective ions used for quantification across all samples, the most prevalent compound was ethyl acetate (RT: 2.74 min, *m*/*z*: 88), an ester with a median peak area of 372,929, which plays a major role in the samples’ distinctive fruity aroma. Among the terpenoids, α-pinene (RT: 10.04 min; *m*/*z*: 93, 187,159) was the most abundant monoterpene, whereas α-humulene (RT: 25.47 min; *m*/*z*: 93, 16,292) was the leading sesquiterpene, both of which are known for their notable bioactivity. The most abundant alcohol identified was 2-methyl-3-buten-2-ol (RT: 2.80 min; *m*/*z*: 71, 67,187), while hexanal (RT: 6.04 min; *m*/*z*: 71, 157,322) was the predominant aldehyde, likely responsible for green and grassy aroma notes. The ketone 2-butanone (RT: 2.64 min; *m*/*z*: 71, 541,830) and the carboxylic acid acetic acid (RT: 2.76 min; *m*/*z*: 60, 298,117) were also detected at high abundance, possibly affecting the resin’s preservative and sensory characteristics. Among hydrocarbons, octane (RT: 6.12 min; *m*/*z*: 43, 186,907) and toluene (RT: 5.20 min; *m*/*z*: 43, 160,412) served as the most prominent aliphatic and aromatic compounds, respectively. These hydrocarbons could originate from the waxes found in propolis, which are known to improve the sealing ability [55]. While diterpenes appeared at lower intensities, abietatriene (RT: 32.01 min; *m*/*z*: 173, 260) was the most abundant in that group. These results reveal a chemically diverse profile dominated by monoterpenes and sesquiterpenes, with potential ecological and pharmacological relevance [56,57]. Our findings are consistent with those of a study conducted across three regions of South Africa, which identified monoterpenes using comprehensive headspace two-dimensional gas chromatography coupled with time-of-flight mass spectrometry (GC × GC–ToF–MS). Notably, key constituents reported in that study included β-pinene, limonene, *p*-cymene, and camphene [58]. Furthermore, a study conducted in Ethiopia on the essential oil of propolis obtained by hydro distillation reported the presence of major compounds such as monoterpenes (e.g., limonene) and sesquiterpenes (e.g., epizonarene and cis-calamenene), which were also identified in the present study [59].

Our findings also resemble a recent study on Algerian propolis, with notable overlap in the major volatile composition. Some of the shared constituents include monoterpenes such as tricyclene, camphene, β-pinene, and eucalyptol, as well as sesquiterpenes such as α-muurolene, trans-calamenene, α-copaene, β-bourbonene, and (E)-caryophyllene. However, Ugandan propolis exhibits a distinctive sesquiterpene profile, including unique components such as cis-14-nor-muurol-5-en-4-one, epizonarene, cadalene, and β-acoradiene, suggesting a specific chemical composition [60]. The TIC for volatile constituents (Figure 2) revealed α-pinene (RT: 10.04 min), a monoterpene, as a common marker in all propolis samples. It was notably abundant in Australian and Uruguayan propolis, as identified by SPME-GC-MS chemical profiling [22]. Additionally, compounds such as α-copaene were detected exclusively in Australian propolis, which is recognised for its high terpenoid content [22].

Generally, the separation order followed the elution of aldehydes, then monoterpenes, followed by sesquiterpenes and diterpenes. Interestingly, the Mbarara samples contained several diterpenes, including neocembrene (cembrene A), which distinguishes them from the other propolis samples (Figure 2A). Neocembrene (RT = 31.68 min) has previously been identified as a diterpene in Saudi Arabian propolis, with predictions indicating that the Cupressaceae and Pinaceae botanical families are its probable sources [61]. The detection of these compounds in Mbarara propolis indicates considerable pharmacological potential and warrants further investigation into their bioactivity and botanical origins. For example, diterpenes have been reported to possess anticancer, antimicrobial, and anti-inflammatory properties [61].

**Figure 5:**
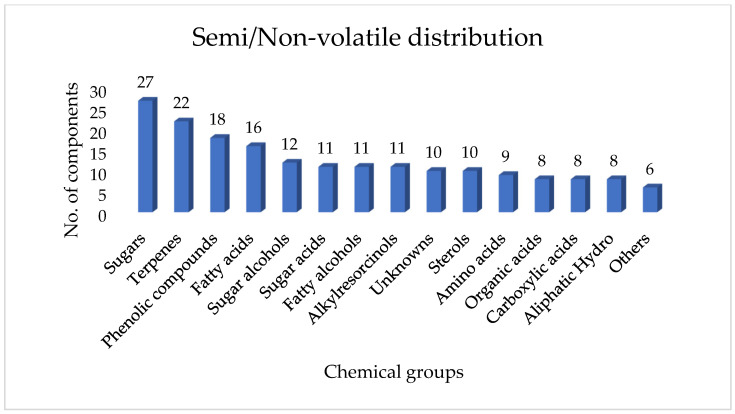
Distribution of the non-volatile composition of Uganda propolis grouped in different phytochemical classes.

A total of 169 chemically distinct compounds were categorised into specific chemical classes (Table S2, in the Supplementary File S2). The representation percentages for each class were identified as follows: phenolic compounds, sugar acids, sugars, glycosylated derivatives, sugar alcohols, organic acids, fatty acids, fatty alcohols, terpenes, aliphatic hydrocarbons, carboxylic acids, amino acids, resorcinols, sterols, and other unknown compounds (Figure 5).

Alanine was the most abundant amino acid (RT: 9.37 min; *m*/*z*: 71, 61,693), presumably due to protein breakdown or the presence of pollen [62]. Among organic acids, a compound featuring a spectrum similar to 2-keto-gluconic acid (RT: 23.07 min; *m*/*z*: 194, 111,100) was predominant, possibly originating from plant or microbial processes. Saccharic acid (RT: 27.15 min; *m*/*z*: 73, 1,510,265) was the dominant sugar acid, while glycerol (RT: 13.20 min; *m*/*z*: 73, 2,154,033) was the most abundant sugar alcohol, indicating a high carbohydrate and lipid content in the resin. Among phenolic compounds, quinic acid (RT: 24.08 min; *m*/*z*: 245, 2,745,723) was the most prominent, a precursor of compounds such as caffeoylquinic acid, which is well-known for its antioxidant properties [63]. The primary fatty acid was octadecenoic acid, 9-(Z)-, oleic acid, (RT: 30.31 min; *m*/*z*: 75, 400,903), and dotriacontanol (RT: 45.55 min; *m*/*z*: 193, 543,534) was the main fatty alcohol, both of which contributed to the lipid profile of propolis. Sugars showed fructose (RT: 24.69 min; *m*/*z*: 307, 1,821,890) as the most abundant. In the group of carboxylic acids, malic acid (RT: 17.80 min; *m*/*z*: 75,604,851) exhibited the highest intensity. Among aliphatic hydrocarbons, n-heneicosane, (RT: 28.61 min; *m*/*z*: 73, 117,402) was the dominant compound, whereas Bilobol C15:1 (RT: 36.65 min; *m*/*z*: 180, 743,253) was the leading alkylresorcinol. The terpenoid profile was diverse, with lupeol (RT: 44.96 min; *m*/*z*: 218, 1,215,658) as the most abundant triterpene. The primary sterol detected was an unknown sterol (RT: 43.50 min; *m*/*z*: 57, 293,338). Other compounds included phosphoric acid, uric acid, guanine, etc., which may not be directly associated with propolis but rather may come from the bees’ diet and metabolic byproducts of their tissues. In summary, the results from the derivatised propolis reveal a chemically diverse composition. Among the identified compounds, triterpenoids, especially resin acids such as pimaric acid, isopimaric acid, and dehydroabietic acid, are reported to originate from resin exudates produced by coniferous species that bees visit [20]. The fatty acids identified in our analysis may originate from beeswax, a key component of propolis. A pyrolysis GC-MS study on derivatised beeswax detected three of our reported fatty acids, i.e., hexadecanoic acid at RT = 28.14 min, hexacosanoic acid at RT = 37.04 min, and tetracosanoic acid at RT = 35.62 min [64]. The total ion current (TIC) for non-volatiles (Figure 3) also showed that sugars, their derivatives, and organic acids are quite abundant in Ugandan propolis. While these compounds may not be our primary focus, their presence could be attributed to honey residues [65].

### 2.4. Multivariate Chemometric Analysis of Propolis Samples from Uganda

The peak intensities of both volatile and non-volatile compounds were analysed using various multivariate statistical methods, including PLS-DA, oPLS-DA, VIP scores, and hierarchical clustering visualisation. These approaches aimed to provide a clearer overview of variability among samples, identify key discriminatory compounds, and reveal overall compositional trends across districts representing diverse agroecological zones in Uganda.

#### 2.4.1. PLS-DA for Geographic Discrimination of Propolis Samples

**Figure 6:**
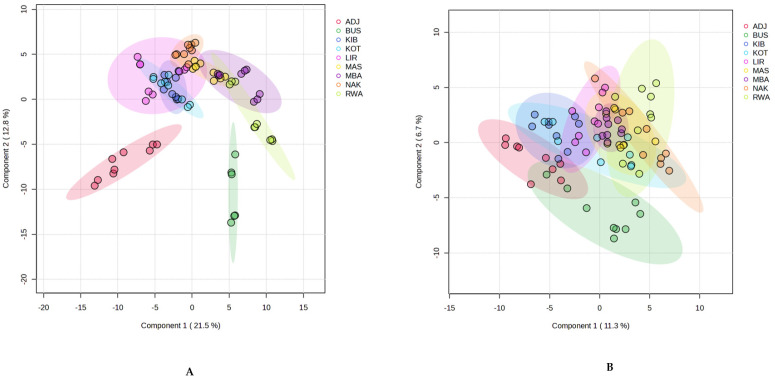
PLS-DA score plots: (**A**): Components 1 and 2 account for 21.5% and 12.8% of total variance in volatiles; (**B**): components 1 and 2 represent 11.3% and 6.7% of total variance in non-volatiles.

The purpose of applying PLS-DA as a supervised technique in this study was to understand if samples could be differentiated by location based on their chemical composition by maximising group separation [66]. The model showed a clear separation between classes by district for the components, with distinct sample clustering along the first two principal components, illustrated in Figure 6.

The score plot for volatile components (Figure 6A), combined 34.3% of the variance within the first two components (Component 1 = 21.5%; Component 2 = 12.8%), with Adjumani (ADJ) and Kotido (KOT) positioned at the opposite ends of Component 1, while Nakasongola (NAK) and Bushenyi (BUS) mainly separated along Component 2. Other samples, Masindi (MAS), Kibuku (KIB), Lira (LIR), Mbarara (MBA) and Rwampara (RWA), clustered near the centre with some overlap. Replicates within districts remained close, demonstrating reproducible volatile profiles. Again, the PLS-DA score plot based on non-volatile constituents explained only 18.0% of the variance in the first two components (Component 1, 11.3%; Component 2, 6.7%) (Figure 6B). Adjumani (ADJ) was on the negative side of Component 1, while Bushenyi (BUS) clustered downward along the negative side of Component 2. Kotido (KOT) and Nakasongola (NAK) showed some separation, though they overlapped with others. MAS, KIB, LIR, MBA, and RWA (with the greatest tendency) mostly overlapped in the centre. Despite overlaps, replicates within each district clustered tightly, indicating consistent non-volatile profiles.

The two PLS-DA models showed that volatile constituents distinguished Ugandan propolis samples more effectively, clearly separating ADJ, KOT, NAK, and BUS. Non-volatiles showed less variance between groups, although ADJ and NAK remained distinct. For example, non-volatiles exhibited less variance between the groups, although several pairs within the groups were separated (e.g., ADJ-NAK, ADJ-RWA). PLS-DA has been used in other studies to highlight the influence of geographical factors on propolis composition [67]. In Uganda, the agroecological zones are grouped based on specific climatic factors such as temperature, rainfall, and altitude, shaping the flora available to bees for resin collection, which plays a pivotal role in determining the composition of propolis, as different plant species produce varying resins and bioactive compounds, which ultimately contribute to the chemical profile of the propolis collected by honeybees [44,45,68]. For instance, Bushenyi district, located in the Western Rangelands, is characterised by vegetation zones comprising moist evergreen forests, savanna woodlands and grasslands with a bimodal rainfall pattern [69] was associated with propolis samples containing a high intensity of monoterpenes reflecting the dominant plant families such as Asteraceae, Rubiaceae, Euphorbiaceae, and Myrtaceae, with various botanical species such as *Eucalyptus camaldulensis* Dehn, *Coffea canephora* Pierre ex Froehner, among others, as bee forage [70,71,72]. Furthermore, sesquiterpenes such as α-copaene, β-bourbonene, and γ-muurolene may have originated from the essential oils of resin-producing plants present in the region, which are used for various agricultural purposes, including *Ficus natalensis* Hochst. and *Albizia coriaria* Welw., *Mangifera indica* L. [73,74].

Conversely, components (volatile and non-volatile) in propolis from Adjumani (West Nile farmlands) formed a distinctly strong separation along the negative axes of component 1 in both score plots (Figure 6). This zone has a distinct semi-arid climate, characterised by arid savanna grasslands interspersed with sparse woodlands and shrubs, highlighting the diversity of resin and gum sources from savannah-type vegetation, with dominant woody species and aromatic trees such as *Balanites aegyptiaca* (L.). Delile and their contributions to propolis composition [75].

**Figure 7:**
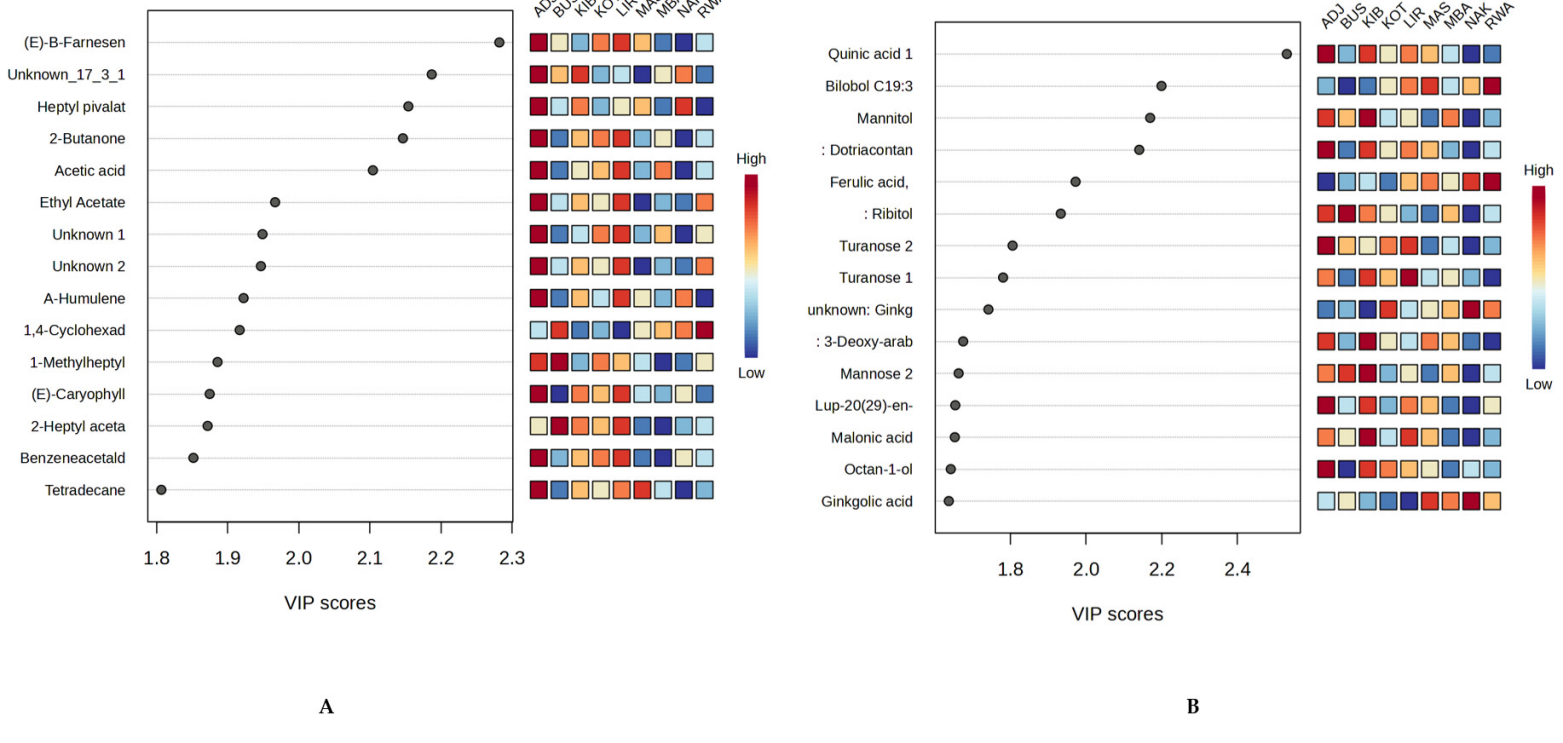
VIP scores for the PLS-DA component 1 highlight the top volatile compounds (VIP > 1.0) for sample classification: (**A**): volatiles and (**B**): non-volatiles.

To assess the impact of both volatiles and non-volatile compounds and their relevance to sample discrimination, VIP scores were calculated for component 1 of the PLS-DA models, using fifteen (15) components with significant VIP values > 1.00, confirming their importance for group separation. For volatiles (Figure 7A), (E)-β-farnesene (RT: 24.60 min, *m*/*z*: 161, VIP = 2.3), Unknown_17_3_112 (RT: 17.29 min, *m*/*z*: 71, VIP = 2.2), heptyl pivalate (RT: 19.84 min, *m*/*z*: 57, VIP = 2.1), 2-butanone (RT: 2.64 min, *m*/*z*: 43, VIP = 2.1) and acetic acid (Rt: 2.76 min, *m*/*z*: 60, VIP = 2.1), with high abundance in Adjumani samples were ranked among the top components significantly contributing to the separation. On the other hand, the non-volatiles displayed a few of the top discriminating components (Figure 7B); these included quinic acid (RT: 24.08 min, *m*/*z*: 245, VIP = 2.5) with high abundance in Adjumani and Kibuku, Bilobol C19:3 (RT: 40.70 min, *m*/*z*: 117, VIP = 2.2) highly abundant in Rwampara and Masindi, mannitol (RT: 25.71 min, *m*/*z*: 217, VIP= 2.0), dotriacontanol (RT: 45.55 min, *m*/*z*: 193, VIP =2.1) with similar abundance like quinic acid, and ferulic acid (RT: 28.42 min, *m*/*z*: 172, VIP= 2.0) which highly abundant in Rwampara and Nakasongola. These findings could help in identifying district or zone-specific chemical markers supporting the notion that ecological reasons are behind the chemical composition of propolis [15].

#### 2.4.2. Heatmap Visualisation of Selected Bioactive Secondary Metabolites

**Figure 8:**
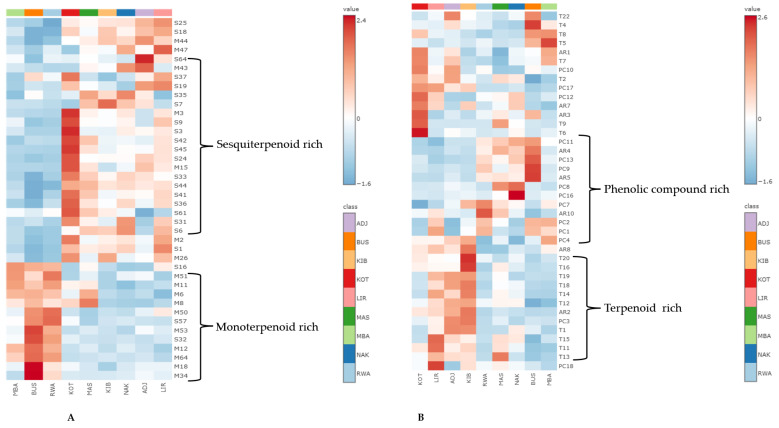
Shows heatmaps of the top 40 bioactive secondary metabolites (monoterpenes and sesquiterpenes) and only terpenes, alkylresorcinols and phenolic compounds from the non-volatile dataset of the propolis samples. Rows represent compounds; columns represent samples grouped by district. Colour intensity indicates relative abundance (normalised scale). (**A**): volatiles and (**B**): non-volatiles, Key: M: Monoterpene, S: Sesquiterpene, T: Terpene, PC: Phenolic compound, AR: Alkylresorcinol.

To further explore the distribution patterns of key metabolites, a heatmap (Figure 8) was created using normalised data on the monoterpene and sesquiterpene composition for the volatile composition and for the terpenes, alkylresorcinols and phenolic compounds from the non-volatile dataset. This chemometric tool is used mainly to illustrate the relative abundances of metabolites, which helps in identifying chemical markers for each geographical location [58].

The heatmap of volatile metabolites (Figure 8A) showed clear, distinct-specific clustering and distinct patterns in the distribution of monoterpenoid (M) and sesquiterpenoid (S) compounds. Bushenyi (BUS) samples were notably rich in monoterpenes, especially *p*-cymenene (M34), pseudolimonene (M18), bornyl acetate (M64), terpinen-4-ol (M53), and umbellulone (M50). Similar, though less pronounced, monoterpene enrichment was observed in Rwampara (RWA) and Mbarara (MBA) samples, indicating that propolis from these districts, spanning different ecological zones, can be distinguished by a monoterpene-rich profile, making these compounds potential regional markers. Conversely, Kotido (KOT) samples showed a strong sesquiterpene enrichment, particularly β-bourbonene (S9), cyclosativene (S3), nerolidol (S42), germacrene B (S45), α-amorphene (S24), δ-amorphene (S41), cadalene (S61), and α-muurolene (S36). This sesquiterpene pattern was also detected, at lower levels, in Masindi (MAS), Kibuku (KIB), Nakasongola (NAK), and Lira (LIR). The repeated presence of these compounds across districts suggests they are useful volatile markers for multiple agroecological zones. These findings are consistent with similar research on propolis from countries like Ethiopia [76], in which propolis from subtropical regions tends to be dominated by monoterpenes, whereas propolis from arid areas with a Mediterranean climate, such as Morocco, often has a higher share of sesquiterpenes [40].

On the other hand, selected non-volatile components (Figure 8B) revealed distinct regional distribution patterns. Samples from Lira (LIR), Adjumani (ADJ) and Kibuku (KIB) demonstrated a relatively similar chemical space consisting of phenolic compounds, such as isovanillic acid (PC18), quinic acid (PC3) and *p*-coumaric acid (PC4), alkylresorcinols, e.g., bilobol C15:0 (AR2), bilobol C17:0 (AR8) and triterpenoids, lanosterol (T11),parkeyl acetate (T13), cycloartenol (T15), eremophilene (T1), α-amyrin (T12), lupeol (T14), lupeol (T18), lupeol acetate (T19), α-bisabolol (T20), lanosterol acetate (T16). These chemical profiles could originate from botanical sources widely distributed in the savannah woodlands and grasslands in both the West Nile and the northern and eastern regions of Uganda [77,78]. Districts located in the western region of Uganda, i.e., RWA (Rwampara), MAS (Masindi) and BUS (Bushenyi), had similar phenolic compound profiles such as cardanol C17:1 (PC9), ginkgolic acid 17:3 (PC13), and hydroginkgolic acid (PC11), with two alkylresorcinols, e.g., bilobol (C17:3, AR5 and C17:2, AR4). Surprisingly, the same chemical pattern was observed in the samples from NAK (Nakasongola). The evidence of bees collecting resins from different botanical species was observed by the presence of diterpene resin acids such as dehydroabietic acid (T5), isopimaric acid (T8) in samples from Bushenyi, Mbarara and KOT (Kotido), which may indicate a similarity in the resin foraging areas for bees in the three regions. This pattern of regional differentiation aligns with findings from other African propolis studies, highlighting the influence of local flora and environmental conditions on the chemical composition [79]. The observed variability highlights the benefit of developing chemotype-based classification systems, which could provide a more rational basis for the pharmacological evaluation and application of propolis.

#### 2.4.3. Geographic Chemometric Differentiation of Ugandan Propolis Using oPLS-DA

Despite the use of PLS-DA in Section 2.4.1, the separation was not that perfect with observed overlaps among samples along the two components; thus, to have a better separation, we performed oPLS-DA analysis on pairwise samples with cross-validation [80]. To analyse geographic variations in the chemical makeup of Ugandan propolis, orthogonal partial least-squares discriminant analysis (oPLS-DA) was performed separately on datasets of volatile and non-volatile compounds (Table S3, in Supplementary File S3). Model evaluation was based on three main metrics: the goodness-of-fit statistic (R^2^Y), the cross-validated predictive ability (Q^2^), and the T score, which indicates the variance explained by the predictive component. Additionally, permutation tests (*n* = 20) were conducted to verify the robustness of each model, with corresponding R^2^Y and Q^2^ values reported. Models were classified as strong if they had permutation-validated R^2^Y ≥ 0.90, Q^2^ ≥ 0.85, and T scores ≥ 25%. Moderate models had R^2^Y between 0.75 and 0.89, Q^2^ between 0.65 and 0.84, and T scores from 15 to 24%, while weak models fell below these thresholds. The volatile compounds dataset (Table S3A, in Supplementary File S3) showed several pairwise comparisons with strong discrimination. The most robust model was KIB vs. BUS (R^2^Y = 0.997, Q^2^ = 0.989, T = 55.8%), demonstrating excellent quality and clear group separation. This was followed by MAS vs. MBA (R^2^Y = 0.984, Q^2^ = 0.947, T = 25.0%), as a moderate model, indicating partial class separation with some overlap. The weakest model was MBA vs. RWA (R^2^Y = 0.995, Q^2^ = 0.927, T = 23.8%), which, despite high R^2^Y and Q^2^, showed lower T scores and less distinct group separation. The non-volatiles dataset (Table S3B, in Supplementary File S3) also showed clear geographic discrimination. The most distinct model was ADJ vs. BUS (R^2^Y = 0.997, Q^2^ = 0.973, T = 26.8%), indicating high predictive ability and strong class separation. The moderately robust model was MAS vs. KIB (R^2^Y = 0.972, Q^2^ = 0.894, T = 22.5%), providing enough separation for classification. The weakest model was observed for MBA vs. RWA (R^2^Y = 0.996, Q^2^ = 0.904, T = 13.3%), indicating poor separation despite high R^2^Y values, likely due to similarities in botanical sources. Interestingly, volatiles distinguish other pairs from non-volatiles.

**Figure 9:**
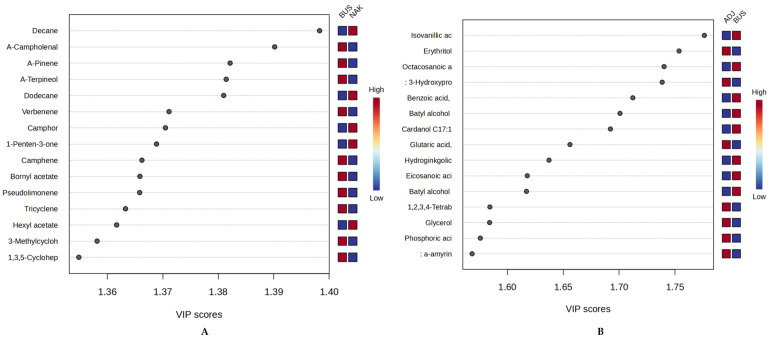
VIP scores for the best oPLS-DA models, both volatiles (**A**) and non-volatiles (**B**).

To identify the discriminating compounds in the excellent models, we conducted a VIP score analysis for NAK vs. BUS (volatiles) and ADJ vs. BUS (non-volatiles), as representative models, as shown in Figure 9 below. For every model, a total of fifteen (15) compounds were considered discriminant (VIP score > 1). Monoterpenes (e.g., α-campholenal, α-pinene, α-terpineol) with high abundance in Bushenyi samples, aliphatic hydrocarbons (e.g., decane) abundantly present in the Nakasongola sample, were observed to be the top discriminating compounds for volatiles with VIP scores > 1.3 (Figure 9A). For non-volatiles, Adjumani samples showed significant abundances of mainly sugar derivatives, such as sugar alcohols (erythritol) and organic acids (3-hydroxypropanoic acid and 2-hydroxyglutaric acid), which could suggest the presence of pollen. In contrast, for Bushenyi samples, the discriminating components are mostly phenolic compounds (e.g., isovanillic acid, *p*-coumaric acid, cardanol C17:1, and hydroginkgolic acid), all with VIP scores > 1.6, as indicated in Figure 9B. These belong to the terpene, resorcinol, and phenolic compound classes, respectively. These findings collectively demonstrate that both volatile and non-volatile chemical profiles can effectively distinguish Ugandan propolis by district, as confirmed by chemometric analysis.

The most reliable and distinctive models were developed for samples from Bushenyi (BUS), Kibuku (KIB), Rwampara (RWA), Adjumani (ADJ), and Kotido (KOT), representing different agroecological zones. The other models, mainly the weaker ones, indicated that some regions might share common factors, such as botanical sources, influencing their chemical composition. Notably, some models linked Mbarara (MBA), Bushenyi (BUS), and Rwampara (RWA) districts, all situated in southwestern Uganda, suggesting they may have quite similar chemical profiles. These findings reinforce the use of multivariate statistical techniques for tracing the chemical origin and chemotaxonomic classification of propolis in Uganda.

## 3. Materials and Methods

### 3.1. Sample Collection and Storage

**Figure 10:**
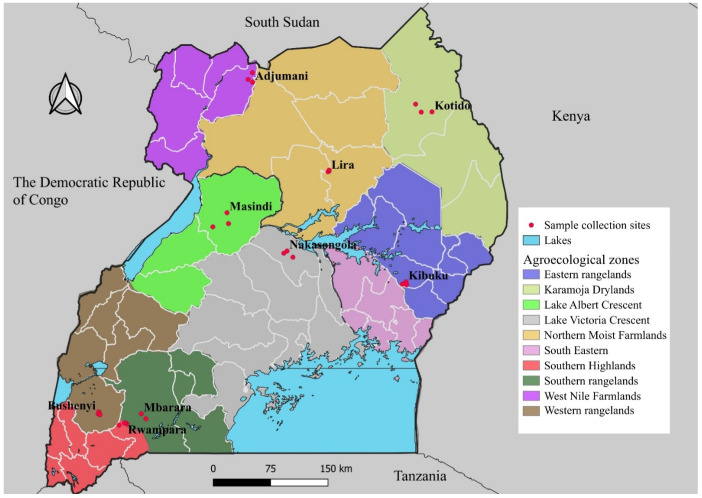
A map showing the districts and their agro-ecological zones visited.

Propolis samples were collected from selected agroecological zones across Uganda between September and October 2024, during the months when beekeepers typically harvest honey. Apiaries in representative districts were chosen based on their active involvement in propolis collection and beekeeping. The selected districts were Nakasongola (NAK) (Lake Victoria Crescent), Masindi (MAS) (Lake Albert Crescent), Bushenyi (BUS) (Western Rangelands), Kibuku (KIB) (Eastern Rangelands), Mbarara (MBA) (Southern Rangelands), Lira (LIR) (Northern Moist Farmlands), Adjumani (ADJ) (West Nile Farmlands), Rwampara (RWA) (Southwestern Highlands), and Kotido (KOT) (Karamoja Drylands). Furthermore, three locations from each district were considered; the GPS coordinates for the sample sites were recorded and illustrated on a map (see Figure 10). About 200 g of raw propolis was collected and stored separately in a dark polyethene bag and stored at room temperature in a dark, dry, cool place for further analysis. Additionally, the colour of the samples from each location was also documented.

### 3.2. Homogenization of Propolis Samples

Forty (40 g) of each raw propolis sample collected from various locations was frozen at −80 °C overnight to facilitate easier processing. The frozen samples were coarsely crushed with a mortar and pestle. Next, the smaller pieces were frozen in liquid nitrogen to increase brittleness. The frozen propolis was finely ground into a powder under liquid nitrogen using a standard laboratory mill (MF10 Basic, IKA Labortechnik, Staufen im Breisgau, Germany) equipped with a 1 mm sieve to ensure uniform particle size. The resulting fine powder was divided into 20 g portions, placed in Falcon tubes, and stored at 4 °C until further analysis. We selected 3 replicates, which were considered appropriate, because we homogenised the samples prior to analysis.

### 3.3. Gas Chromatography–Mass Spectrometry (GC-MS) Analysis

#### 3.3.1. Headspace GC-MS Analysis

Volatile and semi-volatile compounds were analysed in raw propolis using headspace gas chromatography–mass spectrometry (HS-GC-MS) on an HS-20 QP 2010 GC-MS from Shimadzu (Kyoto, Japan) operated by GC-MS LabSolutions Real-time analysis software version 4.20. One hundred (100) mg of raw propolis powder from each district was weighed (±0.1 mg) into 20 mL clear-glass headspace vials (22.5 mm outer diameter × 75.5 mm height) (*n* = 3). The injection programme started with an equilibration step at 70 °C for 8 min. Subsequently, 1 mL headspace was injected in trap mode at a pressurising gas pressure of 50 kPa into the gas chromatograph with a split ratio of 5, using Helium 5.0 (Alphagaz 1, Air Liquide, Düsseldorf, Germany) as the carrier gas, at a linear velocity of 35.1 cm/sec through an 30 m × 0.25 mm × 0.25 μm (film) Rtx-5MS column (Restek, Bellefonte, PA, USA).

The Tenax^®^ trap was initially cooled to 5 °C to efficiently adsorb the compounds from the sample headspace, then dried for 3 min at 31 kPa before thermal desorption at 300 °C. The gas chromatography (GC) oven temperature programme was as follows: after initial hold at 35 °C for 2 min, temperature was increased by 5 °C/min to 150 °C, and then by 15 °C/min until a final temperature of 300 °C. Electron ionisation was applied at 70 eV and 230 °C for 45 min at a scan range of *m*/*z* 36–500, and the resulting ions were detected according to the instrument’s current tune parameters.

GCMS LabSolutions Postrun software version 4.20 (Shimadzu, Kyoto, Japan) was used for data analysis. Automated peak detection utilised a slope threshold of 800/min to enable comprehensive peak identification. To enhance spectral resolution and compound clarity, peak deconvolution was further performed using AMDIS version 2.73 (NIST, Gaithersburg, MD, USA) with shape, resolution, and sensitivity set to medium. The deconvoluted spectra were matched against both the NIST14 library and an in-house database, using only reverse match factors with a minimum acceptance threshold of 80%. Where multiple matches were found, the highest weighted reverse match was selected, and assignments were further confirmed by comparing the experimental retention indices (RI) with literature or library RI values to ensure consistency between spectral identity and chromatographic behaviour.

For quantification, a compound table was generated, selecting quantifier ions typically the most intense and selective *m*/*z* values for each compound spectrum, along with corresponding qualifier ions to confirm identity. In a high-quality sample, a medium number of peaks were designated as quantifiers. Based on this compound table, with retention times (RT) and compound names, peak areas were automatically integrated, and the final table was exported to Microsoft Excel 2019 (Microsoft Corporation, Redmond, USA) using LabSolutions’ built-in data output functionality, facilitating further statistical analysis and documentation.

#### 3.3.2. GC-MS Analysis of Derivatised Propolis Samples

Powdered propolis samples (5 mg each) were accurately weighed into Eppendorf tubes and placed in a vacuum centrifuge (Eppendorf, Hamburg, Germany) for 2 h to eliminate residual water. Following this dehydration step, the samples were subjected to derivatization [81]. For this, 45 µL of 20 mg/mL methoxyamine hydrochloride in pyridine (Sigma Aldrich, Taufkirchen, Germany), was added to each propolis sample, and the mixtures were sonicated for 10 min to improve dissolution. The samples were then incubated in a Bioshaker iQ (Analytik Jena) at 30 °C for 80 min to facilitate the formation of methoxime derivatives. Subsequently, 82 µL of N,O-bis (trimethylsilyl)trifluoroacetamide (BSTFA, CS Chromatographie Service, Langerwehe, Germany) was added to each sample, and silylation was carried out by shaking the tubes at 40 °C for 30 min. After the derivatisation process, the samples were centrifuged at 12,000× *g* for 10 min using an Eppendorf MiniSpin centrifuge. The resulting supernatant was carefully transferred into autosampler vials for subsequent analysis.

For gas chromatography–mass spectrometry (GC-MS) analysis, 1 µL of the derivatised propolis extract was injected into a 6890 GC coupled to an Agilent 5973N mass-selective detector (single quadrupole, MSD, Agilent Technologies, Santa Clara, CA, USA). Helium 5.0 was used as the carrier gas at a flow rate of 1.2 mL/min. The separation was performed on a DB-5MS UI capillary column (30 m × 0.25 mm × 0.25 µm, Agilent Technologies, Böblingen, Germany). The inlet was maintained at 280 °C in splitless mode. The oven temperature was held at 70 °C for 15 min, followed by a ramp increase of 6 °C/min to a final temperature of 325 °C, which was held for 9 min. The mass spectrometer was operated in electron impact (EI) ionisation mode at 70 eV, with a scan range of *m*/*z* 55–800 for 55 min. Data acquisition and analysis were performed using Agilent MassHunter Workstation (version B.02.00, Agilent Technologies, Waldbronn, Germany).

A new batch was generated by loading the reference data file into Agilent’s MassHunter Quantitative Analysis Software B.07.00, and the method was created by initiating the automated peak picking process. Compound identification was performed by comparing the acquired mass spectra with the NIST08 spectral database (National Institute of Standards and Technology, Gaithersburg, MD, USA), using a minimum match factor threshold >70 to obtain reasonable library hits. The resulting compound list was further refined through manual review and validation of retention times (RTs), with iterative selection of appropriate quantifier and qualifier ions. The optimised method was then applied to the batch, and manual peak integration was carried out where necessary. Quantitative data, including retention time, compound name, and quantifier ion, were subsequently exported to Microsoft Excel 2019 using the “Export Table” function.

### 3.4. Multivariate Data Analysis

The analyte-intensity tables (.csv format) were further processed using the MetaboAnalyst 6.0 web interface, “https://www.metaboanalyst.ca/ (accessed on 25 August 2025)”, for visualisation of the data by heatmap, Partial Least-Squares Discriminant Analysis (PLS-DA), and Variable Importance in Projection (VIP) analysis to identify potential marker compounds within the different propolis samples. Data were median-normalised, square root-transformed, and auto-scaled to optimise model parameters [82]. Supervised (s)PLS-DA was used to classify propolis samples by geographical location, and VIP scores identified significant compounds. The PLS-DA model was validated through 5-fold cross-validation and permutation tests [83]. Model performance was evaluated across five components, and statistical significance was assessed using 2000 permutations, with p-values calculated using the plus-one estimator at α = 0.05. Additionally, an oPLS-DA was performed to identify the most discriminative volatile compounds, distinguishing them across various sample collection sites.

## 4. Conclusions

This study presents the first detailed chemical analysis of Ugandan propolis, for the first time integrating two GC–MS techniques in such a study: headspace GC-MS for volatile compounds and liquid injection GC-MS after derivatisation for non-volatile substances, alongside chemometric modelling. The profiles closely resemble those of tropical propolis, particularly in the presence of terpenoids, including monoterpenes, sesquiterpenes, diterpenes, and triterpenoids. Ugandan propolis exhibits greater diversity and regional specificity in sesquiterpenes and diterpene resin acids than other African samples. Compounds such as neocembrene, bilobol, and several unidentified substances indicate specific regional chemotypes and the potential to discover new bioactive compounds. Volatile compounds were more effective at distinguishing geographic regions, with oPLS-DA models based on volatiles performing better than those based on non-volatiles. Heatmap clustering of monoterpenes and sesquiterpenes revealed clearer district-level signatures than those of non-volatile compounds, suggesting that volatiles are superior regional markers. Nonetheless, non-volatiles, including phenolic acids and triterpenoids, still offered valuable insights across different zones.

Our primary contribution focused on developing a chemometric framework for geographically classifying Ugandan propolis, thereby enhancing traceability and pharmacological prospects. The performance of our models solely relied on the compounds detected; thus, some undetected compounds, such as polar flavonoids, glycosylated compounds, and high-molecular-weight wax esters, may not have been detected with the techniques we employed. We therefore recommend future research to incorporate methods such as LC-MS/MS, pyrolysis-GC-MS, or NMR to explore the full chemical space and improve classification breadth and accuracy. Furthermore, future studies on integrating chemical and biological data will enable linking chemical diversity with ecological factors and bioactivity. In summary, we can confidently affirm that Ugandan propolis is chemically diverse and regionally distinctive, with volatile terpenoid profiles serving as reliable geographic indicators. Our findings provide a strong foundation for advanced chemotaxonomic classification of African propolis, encouraging the expansion of analytical methodologies to fully explore its chemical and biological potential.

## Data Availability

All the details used to generate this manuscript have been provided in the Supplementary Material Files. Datasets used can only be shared upon having a convincing request from the corresponding authors.

## Supplementary Materials

The following supporting information can be downloaded at: https://www.mdpi.com/article/10.3390/molecules30224435/s1, Supplementary File S1: List of volatile chemical compounds. Supplementary File S2: List of non-volatile chemical compounds. Supplementary File S3: oPLS-DA pairwise comparisons for both volatile and non-volatile components.
