# Supplementary material for: Chemical Profiling and Geographic Differentiation of Ugandan Propolis by GC-MS Through Chemometric Modelling
Source: Molecules. 2025 Nov 16;30(22):4435. doi: 10.3390/molecules30224435 (PMC12655498; doi:10.3390/molecules30224435)
Supplement: Supplementary file 1 [file molecules-30-04435-s001.zip › Supplementary file S1.pdf]

Table S 1 Representative list of 216 tentatively identified volatile chemical components (sorted according to their median peak area). Peak deconvolution was carried out with AMDIS 2.73 with shape / resolution / sensitivity set to medium. Search against NIST and FFNSC2 with reverse search at a minimum match of 80. Matches were done based on appropriate spectral matches and nearest RI.

Class	Name	RT [min]	Model	Median Peak area
Alcohols	**3-Buten-2-ol, 2-methyl-**	2.80	**71 *m/z***	**67187**
	2-Methyl-1-propanol	2.89	84 *m/z* (43)	42400
	3-Methylbutanol	4.56	55 *m/z*	53203
	Pentanol	5.28	42 *m/z*	4672
	1-Penten-3-ol, 2-methyl-	5.62	71 *m/z*	5027
	n-Hexanol	8.06	56 *m/z*	10702
	Cyclohexan-1,4,5-triol-3-one-1-carboxylic acid	8.15	56 *m/z*	11577
	3-Hexen-1-ol, (Z)-	8.76	67 *m/z*	14349
	2-Heptanol	9.01	45 *m/z*	331
	2-Methyl-4-penten-2-ol	10.57	97 *m/z*	11303
	p,α-Dimethylbenzyl alcohol	12.13	121 *m/z*	201
	2-Nonanol	15.36	45 *m/z*	348
Aldehydes	3-Methylbutanal	3.24	41 *m/z* (43)	35588
	Pentanal	3.82	44 *m/z*	54985
	3-hexenal	5.03	85 *m/z* (73)	2344
	2-Pentenal	5.67	84 *m/z* (97)	7352
	**Hexanal**	6.04	**44 *m/z***	**157322**
	Benzeneacetaldehyde	10.91	106 *m/z*	96
	Octanal	12.34	43 *m/z*	7966
	Nonanal	15.44	57 *m/z* (71)	441
Aliphatic hydrocarbons	Heptane	3.90	41 *m/z*	49274
	1,4-Cyclohexadiene, 1,2-dimethyl-	4.31	93 *m/z* (44)	158
	**Octane**	6.12	**43 *m/z***	**186907**
	1,3-Cyclopentadiene, 5,5-dimethyl-1-ethyl-	6.40	107 *m/z*	96
	2-Octene, (Z)-	6.44	55 *m/z*	1936
	3,3,5,5-Tetramethylcyclopentene	7.02	109 *m/z* (207)	683
	1,3-Cyclopentadiene, 5,5-dimethyl-2-ethyl-	7.55	109 *m/z*	133
	1,3,5-Cycloheptatriene, 3,7,7-trimethyl-	8.55	119 *m/z*	86
	Nonane	9.08	57 *m/z* (44)	18413
	Norbornane, 2,2-dimethyl-5-methylene-	10.21	79 *m/z* (75)	65
	1-Decene	11.91	70 *m/z* (105)	842
	Decane	12.19	57 *m/z*	16731
	Undecane	15.43	57 *m/z*	9786
	Dodecane	18.36	57 *m/z*	12801
	Tridecane	21.32	57 *m/z* (93)	4673
	Tetradecane	24.00	57 *m/z*	3516
	Pentadecane, 2,6,10,14-tetramethyl-	28.92	57 *m/z*	163
Aromatic hydrocarbons	**Toluene**	5.20	**91 *m/z***	**160412**
	Mesitylene	11.94	105 *m/z* (70)	451
	Benzene, 1-methyl-4-(1-methylpropyl)-	15.59	119 *m/z* (139)	153
	Acetic acid	2.76	60 *m/z*	298117

**Carboxylic acids**	Propanoic acid	3.60	74 *m/z*	64
	Isobutyric acid	4.95	43 *m/z*	6679
**Esters**	**Ethyl Acetate**	2.74	**43 *m/z* (88)**	**372929**
	Isopropyl acetate	3.27	43 *m/z* (41)	52857
	Ethyl propanoate	4.05	57 *m/z*	3944
	Propyl acetate	4.10	43 *m/z*	13920
	Isobutyl acetate	5.40	43 *m/z* (95)	12210
	Ethyl isovalerate	7.58	88 *m/z* (109)	227
	1-Butanol, 3-methyl-, acetate	8.27	43 *m/z* (74)	7833
	Hexenyl butanoate (3Z)	9.42	43 *m/z* (107)	1416
	Hexyl acetate	12.53	43 *m/z*	3206
	2-Heptyl acetate	13.38	43 *m/z*	5778
	1-Octen-3-yl acetate	14.53	128 *m/z* (59)	4133
	2-Octanol, acetate	16.41	43 *m/z* (134)	322
	Butanoic acid, 1-methylhexyl ester	17.26	71 *m/z*	454
	1-Methylhexyl butyrate	18.63	71 *m/z*	768
	endo-Fenchyl acetate	18.86	81 *m/z* (109)	175
	1-Methylheptyl propionate	18.95	57 *m/z*	605
	2-Nonanol, acetate	19.28	87 *m/z*	79
	Heptyl pivalate	19.84	57 *m/z*	2011
	Wax ester	23.84	99 *m/z*	107
**Ketones**	**2-Butanone**	2.64	**43 *m/z***	**541830**
	2-Butanone, 3-methyl-	3.32	43 *m/z* (56)	52857
	2-Acetylfuran 1	5.54	95 *m/z*	2252
	Acetonylacetone	5.78	97 *m/z* (55)	11937
	2-Acetylfuran 2	5.94	97 *m/z* (55)	2132
	3,4-Hexanedione	7.23	57 *m/z*	3057
	2-Cyclopenten-1-one, 3,4,5-trimethyl-	7.44	107 *m/z*	2425
	Heptan-2-one	8.64	43 *m/z*	6086
	3-Methylcyclohexanone	9.96	69 *m/z* (50)	1286
	1-Penten-3-one	11.33	55 *m/z* (43)	581
	2-Octanone	11.92	43 *m/z* (81)	7075
	2-Nonanone, 3-(hydroxymethyl)-	15.11	58 *m/z*	2411
**Monoterpenes**	Cyclofenchene	8.39	93 *m/z*	189
	5-Hexen-2-one, 5-methyl-3-methylene-	8.58	81 *m/z*	3061
	ε-Cyclogeraniolene	8.67	82 *m/z*	12375
	2-Bornene	9.10	93 *m/z* (80)	396
	Hashishene (hit)	9.58	93 *m/z*	489
	Tricyclene	9.66	93 *m/z*	3093
	α-Thujene	9.80	93 *m/z*	1885
	**α-Pinene**	10.04	**93 *m/z***	**187159**
	α-Fenchene	10.48	79 *m/z*	2715
	Camphene	10.53	93 *m/z*	11660
	Thuja-2,4(10)-diene	10.66	91 *m/z*	3600
	Verbenene	11.22	119 *m/z*	444
	Sabinene	11.28	93 *m/z*	491
	β-Pinene	11.42	93 *m/z*	5667

	cis-Pinane	11.57	95 *m/z*	288
	Myrcene	11.83	93 *m/z*	3643
	α-Phellandrene	12.34	93 *m/z* (119)	3056
	Pseudolimonene	12.43	93 *m/z*	3262
	α-Terpinene	12.69	121 *m/z*	2272
	p-Cymene	12.77	119 *m/z*	671
	o-Cymene	12.92	119 *m/z*	18227
	Limonene	13.08	68 *m/z* (57)	7079
	Eucalyptol	13.16	43 *m/z*	5674
	Unknown monoterpene_13_25_119	13.25	119 *m/z* (93)	564
	β-Ocimene	13.30	93 *m/z*	2432
	Lavender lactone	13.36	111 *m/z*	3998
	Unknown monoterpene_13_46_119	13.47	119 *m/z*	97
	(E)-β-ocimene	13.63	93 *m/z*	2434
	γ-Terpinene	14.00	93 *m/z*	4209
	trans-Linalool oxide (furanoid) 1	14.39	59 *m/z* (99)	1915
	m-Cymenene	14.72	132 *m/z*	94
	Terpinolene	14.87	93 *m/z*	2814
	trans-Linalool oxide (furanoid) 2	14.88	59 *m/z* (93)	128
	p-Cymenene	15.00	58 *m/z*	257
	Linalool	15.29	93 *m/z* (107)	1535
	Filifolone	15.31	107 *m/z*	785
	Rose oxide	15.62	139 *m/z* (119)	97
	p-Mentha-1,3,8-triene	15.71	134 *m/z* (109)	134
	β-Thujone	15.85	110 *m/z* (81)	131
	endo-Fenchol	15.90	81 *m/z* (107)	398
	Chrysanthenone	15.96	107 *m/z*	202
	α-Campholenal	16.12	108 *m/z*	274
	Alloocimene	16.18	121 *m/z*	233
	cis-p-Mentha-2,8-dien-1-ol	16.25	138 *m/z*	70
	Hit: *p*-Mentha-1,3,8-triene	16.39	134 *m/z*	132
	Pinocarveol	16.57	92 *m/z*	189
	Camphor	16.74	95 *m/z(122)*	4442
	Camphene hydrate	17.00	93 *m/z*	138
	Pinocamphone	17.16	83 *m/z* (140)	280
	Umbellulone	17.42	108 *m/z*	182
	Borneol	17.51	95 *m/z* (59)	631
	Pinocamphone <cis->	17.62	55 *m/z*	666
	Terpinen-4-ol	17.77	71 *m/z*	430
	α-Terpineol	18.19	59 *m/z*	134
	Myrtenal	18.20	107 *m/z*	468
	Myrtenol	18.21	79 *m/z* (59)	199
	Verbenone	18.55	107 *m/z*	394
	Thymol methyl ether	19.19	149 *m/z*	116
	Carvacrol Methyl Ether	19.46	149 *m/z*	146
	Cuminaldehyde	19.60	135 *m/z* 178	100
	Carvone	19.61	82 *m/z*	159

	Carvenone	19.97	110 *m/z* (82)	118
	Menthone	20.67	111 *m/z* (43)	115
	Bornyl acetate	20.76	95 *m/z*	191
	Thymol	20.91	135 *m/z*	132
	2-ethyl isomenthone	20.98	111 *m/z*	135
	Neoiso-dihydrocarveol acetate	22.46	93 *m/z*	113
	Isomenthone	28.07	43 *m/z*	740
**Sesquiterpene**s	cis-Arbusculone	10.86	93 *m/z*	1635
	α-Cubebene	21.54	161 *m/z*	125
	Cyclosativene	22.64	105 *m/z*	493
	α-Ylangene	22.86	123 *m/z* (159)	114
	β-Panasinsene	23.14	105 *m/z*	1477
	β-Cubebene	23.21	161 *m/z* (107)	1829
	α-Copaene	23.31	105 *m/z*	9635
	β-Longipinene	23.53	81 *m/z* (93)	1733
	β-Bourbonene	23.64	81 *m/z*	1389
	(Z)-caryophyllene	23.66	107 *m/z* (161)	409
	α-Cedrene	23.75	108 *m/z* (193)	387
	cis-Thujopsene	24.06	161 *m/z*	402
	trans-α-Bergamotene	24.11	119 *m/z* (161)	926
	γ-Elemene	24.23	107 *m/z*	695
	γ-Acora-3,7(14)-diene	24.32	136 *m/z*	191
	α-Guaiene	24.40	119 *m/z*	1351
	α-Himachalene	24.48	133 *m/z* (191)	8510
	(E)-Caryophyllene	24.58	93 *m/z*	9714
	(E)-β-Farnesene	24.60	161 *m/z*	3087
	cis-Muurola-4(14),5-diene 1	24.74	161 *m/z* (93)	921
	β-Acoradiene	24.88	123 *m/z*	175
	γ-Muurolene	25.12	161 *m/z*	353
	γ-Curcumene	25.21	119 *m/z*	943
	α-Amorphene	25.21	161 *m/z*	1146
	**α-Humulene**	25.47	**93 *m/z***	**16292**
	cis-Muurola-4(14),5-diene 2	25.52	161 *m/z* (119)	1174
	Epizonarene	25.56	119 *m/z*	1667
	9-epi-Caryophyllene	25.57	91 *m/z*	1910
	Trans-β-Guaiene	25.61	119 *m/z* (281)	969
	α-Cuprenene	25.72	161 *m/z*	2128
	γ-Cadinene	25.78	161 *m/z*	2834
	Ar-Curcumene	25.86	132 *m/z*	200
	trans-Cadina-1,4-diene	26.13	105 *m/z*	2211
	Zonarene	26.20	161 *m/z*	1427
	α-Cadinene 1	26.24	105 *m/z*	5305
	α-Muurolene	26.32	105 *m/z*	7335
	Elemol	26.38	69 *m/z*	662
	Selina-3,7(11)-diene	26.51	161 *m/z*	2345
	Germacrene B 1	26.59	161 *m/z*	4003
	cis-Calamenene	26.63	159 *m/z* (69)	15942

	δ -Amorphene	26.66	161 *m/z*	6032
	Nerolidol	26.66	69 *m/z*	971
	trans-Calamenene	26.71	159 *m/z* (161)	15942
	α-Cadinene 2	26.74	105 *m/z*	4407
	Germacrene B 2	26.83	119 *m/z* (157)	1356
	α-Calacorene	26.85	157 *m/z* (119)	974
	α-Cadinene 3	26.90	105 *m/z* (109)	1437
	Longipinanol	27.22	69 *m/z*	484
	β-Calacorene	27.29	157 *m/z*	251
	Caryophyllene oxide	27.58	41 *m/z*	566
	Gleenol	27.63	121 *m/z*	318
	β-Oplopenone	27.88	177 m/z	96
	epi-Cedrol	27.98	95 *m/z* (177)	124
	Unknown, Hit: cis-Muurola-4(14),5-diene 1	28.03	185 *m/z*	636
	Unknown,Hit: cis-Muurola-3,5-diene	28.19	119 *m/z* (159)	176
	α-Acorenol	28.26	119 *m/z*	151
	Unknown,Hit: trans-Cadina-1(6),4-diene	28.34	161 *m/z*	167
	Unknown, Hit:Longipinanol	28.35	105 *m/z*	297
	Unknown, Hit: cis-Muurola-4(14),5-diene 2	28.42	159 *m/z* (43)	120
	epi-A-Muurolol	28.51	161 *m/z*	184
	Cadalene	28.70	183 *m/z*	2866
	α-Bisabolol	28.84	69 *m/z* (164)	205
	cis-14-nor-Muurol-5-en-4-one	28.87	164 *m/z*	137
	10-Nor-calamenen-10-one	29.04	159 m/z	132
	Oplopanone	29.35	135 m/z	90
**Diterpenes**	Hit: Isopimara-9-(11),15-diene	31.01	257 *m/z* (241)	100
	Hit: Sandaracopimara-8(14),15-diene 1	31.28	137 *m/z* (83)	139
	Hit: Sandaracopimara-8(14),15-diene 2	31.42	137 *m/z* (109)	152
	Neocembrene	31.68	81 *m/z*	184
	13-epi-Manoyl oxide	31.77	257 *m/z* (159)	109
	**Abietatriene**	**32.01**	**173 *m/z***	**260**
	Abietadiene	32.26	229 *m/z* (200)	71
**Unknowns**	Unknown 1	2.70	45 *m/z* (82)	181201
	Unknown 2	2.79	43 *m/z*	371162
	Unknown 3	2.89	84 *m/z* (43)	703
	Unknown 4	2.95	41 *m/z* (67)	42400
	Unknown 5	3.69	43 *m/z*	14514
	Unknown 6	4.10	43 *m/z* (45)	13920
	Unknown 7	4.67	69 *m/z* (79)	4445
	Unknown 8	4.68	79 *m/z* (57)	2567
	Unknown 9	4.85	95 *m/z*	248
	Unknown 10	4.93	57 *m/z*	11111
	Unknown 11	5.48	43 *m/z* (95)	12900
	Unknown 12	6.83	107 *m/z*	106
	Unknown 13	7.70	43 *m/z*	750
	Unknown 14	8.03	119 *m/z*	84
	Unknown 15	8.84	105 *m/z*	829

	Unknown 16	9.91	119 *m/z*	145
	Unknown 17	10.35	91 m/z	249
	Unknown 18	11.61	99 *m/z* (60)	2553
	Unknown 19	12.31	119 *m/z* (134)	397
	Unknown 20	14.46	99 *m/z* (59)	7323
	Unknown 21	15.73	109 *m/z* (43)	528
	Unknown 22	16.07	57 *m/z*	714
	Unknown 23	17.29	112 *m/z* (71)	109
	Unknown 24	20.05	(71)	10479
	Unknown 25	20.46	108 *m/z*	138
	Unknown 26	21.68	57 *m/z* (101)	451
	Unknown 27	22.53	105 *m/z* (85)	365
	Unknown 28	24.06	204 *m/z*	212
	Unknown 29	25.37	93 *m/z*	12974
	Unknown 30	26.43	132 *m/z*	170
	Unknown 31	27.30	81 *m/z*	993
	Unknown 32	28.16	159 *m/z* (43)	141
	Unknown 33	31.09	81 *m/z*	203
