# Supplementary material for: Chemical Profiling and Geographic Differentiation of Ugandan Propolis by GC-MS Through Chemometric Modelling
Source: Molecules. 2025 Nov 16;30(22):4435. doi: 10.3390/molecules30224435 (PMC12655498; doi:10.3390/molecules30224435)
Supplement: Supplementary file 1 [file molecules-30-04435-s001.zip › Supplementary file S2.pdf]

Table S2 Retention time (RT), Kovac Index (RI), ion traces for quantification (quan ion) and medium intensity of 177 tentatively identified or characterized non-volatile compounds in derivatised Ugandan propolis samples. Identification parameters were set to a minimal match factor of 80 and use of RI data to match the expected relative retention times. Deconvolution was carried out with one adjacent peak subtraction, medium resolution and sensitivity, and low shape requirements. ~ 1300 signals were detected, most of those close to the background with insufficient spctral quality but indicating the compositional richness of the samples. ":" means NIST library hit >800 reverse match with inappropriate RI or manual compound type annotation based on spectral similarity.

Class	Compound Name	RT [min]	RI	Model	Median Peak Area
**Amino Acids**	Alanine	9.37	1124.0	116 m/z (71)	61693
	Valine	11.87	1217.8	144 m/z (55)	3741
	Proline	13.69	1290.9	142 m/z	2732
	Glycine	13.87	1298.6	174 m/z (341)	5564
	:N-Methyl-L-leucine	16.76	1425.1	73 m/z (172)	15489
	Pyroglutamic acid	18.43	1503.2	156 m/z (132)	26637
	: Proline	19.65	1563.6	142 m/z (217)	1364
	unknown_23_01_217: Penicillamine	23.01	1741.7	217 m/z	87500
	Tyramine	25.36	1878.0	73 m/z	24012
**Organic acids**	Lactic acid	8.45	1091.1	191 m/z	44598
	Glycolic acid	8.80	1103.5	177 205	37281
	Pyruvic acid	9.04	1111.9	147 m/z	13429
	: 3-Hydroxypropanoic acid	10.30	1157.9	147 m/z (71)	682434
	Malonic acid	11.63	1208.4	147 m/z	35454
	Glutaric acid, 2-hydroxy-	19.56	1558.7	129 m/z	7272
	**: 2-Keto-L-gluconic acid**	**23.07**	**1652.0**	**297, 312**	**111100**
	: phenylpropanoic acid similar to 4-Hydroxy-3-methoxyphenylglycol	27.01	1980.3	73 m/z (289)	1421
**Sugar Acids**	Glyceric acid	14.42	1321.9	73 m/z (97)	29353
	Threonic acid-1,4-lactone	16.50	1412.9	147 m/z	12090
	Threonic acid	18.89	1525.8	73 m/z (211)	319917
	Glucuronic acid 1	25.65	1895.5	73 m/z (174)	9686
	Glucuronic acid 2	25.98	1916.0	319 m/z (333)	42779
	Glucaric acid-1,4-lactone	26.09	1922.8	293 m/z	9948
	Gulonic acid	26.35	1938.8	281 m/z	3843
	Gluconic acid	26.73	1962.6	204 m/z	59071
	Galactonic acid	26.94	1976.2	73 m/z	698304
	**Saccharic acid**	**27.15**	**1989.2**	**297 m/z (73)**	**1510265**
	: 3-Deoxy-arabino-hexaric acid	28.71	2091.4	57 m/z	11782
**Sugar Alcohols**	**Glycerol**	**13.20**	**1270.9**	**73 m/z**	**2154033**
	1,2,3,4-Tetrabutanol	13.62	1288.2	73 m/z	2030072
	Erythritol	18.20	1492.2	73 m/z (93)	1789988
	Xylitol	22.26	1700.0	73 m/z (143)	435009
	Arabitol	22.34	1704.6	73 m/z	109117
	Mannitol	25.71	1899.6	333 m/z (217)	363516
	Sorbitol	25.94	1913.3	275 m/z (319)	16808
	Inositol, scyllo-	27.18	1990.9	73 m/z	1562775

	: Ribitol	27.45	2008.8	318 m/z	12420
	Inositol, myo-	27.83	2033.5	117 m/z (280)	5094
	unknown_32_93_204: sugar alcohol or glycerol pyranoside	32.81	2385.4	239 m/z	9333
	Maltitol	38.47	2861.5	117 m/z	597428
**Phenolic compounds**	Benzoic acid, 4-hydroxy-	20.56	1609.8	267 m/z (204)	53054
	Salicylic acid	21.70	1670.0	267 m/z	35250
	**Quinic acid 1**	**24.08**	**1802.6**	**245 m/z**	**2745723**
	Cinnamic acid, 4-hydroxy-	26.01	1917.5	333 m/z (319)	48154
	Gallic acid	26.21	1929.9	73 m/z	144751
	Ferulic acid, cis-	28.42	2072.0	73 m/z (174)	4584
	Caffeic acid, cis-	29.01	2111.5	352 m/z	6448
	Quinic acid 2	35.97	2641.0	361 m/z	6135
	Cardanol C17:1	36.54	2689.6	205 m/z	10584
	Ginkgolic acid 15:1	38.23	2839.8	204 m/z	25402
	Hydroginkgolic acid	38.37	2852.1	475 m/z	25202
	unknown: Ginkgolic acid 17:2	39.23	2932.0	57 m/z (268)	36756
	unknown: Ginkgolic acid 17:3	39.67	2973.7	439 m/z	19624
	Ginkgolic acid 17:1, minor	40.34	3038.9	426, 501 m/z	3742
	Ginkgolic acid 17:1, main	40.45	3048.8	424, 499	4513
	Ginkgolic acid 17:0	40.65	3068.9	428, 503 m/z	7426
	phenolic acid derivative_46_85_267	46.65	3720.9	117, 537, 552	14441
	Isovanillic acid	49.77	4113.5	441 m/z 467 495 510 585	13671
**Fatty Acids**	Octanoic acid	13.02	1263.5	201 m/z	2732
	Mevalonic acid-1,5-lactone	15.27	1358.5	145 m/z (117)	1877
	Hexadecanoic acid	27.50	2012.0	204 m/z (279)	13787
	Octadecenoic acid methyl ester, 9-(Z)-	29.53	2147.1	327 m/z	4500
	Octadecadienoic acid, 9,12-(Z,Z)-	29.70	2158.8	69 m/z	29898
	**Octadecenoic acid, 9-(Z)-**	**30.31**	**2201.6**	**75 m/z**	**400903**
	Octadecanoic acid	30.41	2208.9	73 m/z	179771
	unknown_31_84_117: fatty acid derivative	31.65	2298.3	57 m/z	7079
	Eicosanoic acid	33.26	2420.3	256 m/z (462)	3229
	Tetracosanoic acid	38.45	2859.3	477 m/z	9130
	Hexacosanoic acid	40.54	3058.2	428, 503	34223
	unknown_41_34_117: fatty acid derivative	41.07	3110.4	268, 514	3208
	Octacosanoic acid	42.68	3275.5	393 m/z (527)	19095
	Triacontanoic acid	44.72	3497.4	393 m/z (509)	95474
	unknown_46_65_117: fatty acid derivative	46.20	3668.1	75 m/z (189)	4871
	: fatty acid pyranoside	51.77	4386.8	204 m/z	1731

**Fatty alcohols**	Octan-1-ol	9.42	1125.6	187 m/z	2953
	: 2-Methyl-1,3-butanediol	15.42	1364.7	73 m/z	11064
	Octadecan-1-ol	29.20	2124.3	219 m/z	156089
	Batyl alcohol 1	36.30	2668.7	345 m/z	12328
	: 1-Tetracosanol	37.36	2761.1	204 m/z	84028
	Batyl alcohol 2	38.79	2891.2	361 m/z	23881
	: 1-Hexacosanol	39.20	2929.1	268 m/z	5369
	: Octacosanol	41.34	3137.8	117 m/z	3532
	: 1-Triacontanol	43.80	3396.0	109 m/z	39171
	**: Dotriacontanol**	**45.55**	**3591.9**	**199 m/z (193)**	**543534**
	: Tetratriacontanol (1TMS)	47.65	3842.1	509 m/z 524	70421
**Sugars**	Glyceraldehyde	12.00	1222.7	73 m/z (227)	6965
	Erythrose	17.08	1439.4	73 m/z	74788
	Ribose	21.20	1643.1	73 m/z	63345
	Arabinose	21.48	1658.2	103 m/z	19431
	Rhamnose	22.19	1696.4	117 m/z (143)	59887
	Fucose	22.19	1696.4	117 m/z (143)	59887
	**Fructose 1**	**24.69**	**1838.1**	**73 m/z (307)**	**1821890**
	Fructose 2	24.94	1852.8	73 m/z (349)	1033655
	Glucose 1	25.10	1862.5	73 m/z	660167
	Glucose 2	25.15	1865.7	73 m/z	520580
	Mannose 1	25.23	1870.5	73 m/z (245)	1051277
	Mannose 2	25.62	1893.7	174 m/z (73)	397660
	unknown_26_68_217: furanose	26.48	1946.7	73 m/z (160)	5554
	Glucopyranose_26_73_204	26.68	1959.7	217 m/z (204)	41659
	unknown_33_67_204: pyranoside	33.54	2442.3	117 m/z	140836
	Sucrose	35.36	2597.0	445 m/z	142554
	unknown_37_04_361: disaccharide	36.89	2720.1	57 m/z	65569
	Maltose 1	37.04	2733.2	361 m/z	39922
	Trehalose, alpha,alpha'-	37.17	2744.8	204 m/z	79327
	Turanose 1	37.26	2752.1	361 m/z (411)	136972
	Maltose 2	37.39	2764.2	411 m/z	156491
	unknown_37_61_361: disaccharide	37.51	2774.2	361 m/z (397)	4540
	Turanose 2	37.61	2783.1	361 m/z	17718
	: Sophorose 1	37.69	2790.4	73 m/z	17924
	: Sophorose 2	38.00	2818.2	268, 490	18172
	: Gentiobiose	38.00	2818.3	217 m/z (57)	532039
	Isomaltose	38.78	2889.6	268 m/z, 488	23532
**Carboxylic acids**	Benzoic acid	12.64	1248.3	179 m/z	15612
	Succinic acid	14.10	1308.3	147 m/z	168134
	Fumaric acid	14.92	1342.9	245 m/z	28977
	**Malic acid**	**17.80**	**1473.4**	**73 m/z**	**604851**
	unknown_23_07_165: 4-Cyclohexene-1,2-dicarboxylic acid	23.07	1744.8	165 m/z (319)	5122

	Shikimic acid	23.21	1752.8	73 m/z (160)	164604
	Citric acid	23.92	1793.1	204 m/z (244)	540563
	Isocitric acid	24.03	1799.6	73 m/z	506769
**Aliphatic Hydrocarbons**	**Heneicosane, n-**	**28.61**	**2084.7**	**73 m/z**	**117402**
	Tricosane	31.54	2290.1	204 m/z (175)	29934
	Pentacosane	33.67	2452.7	204 m/z	8894
	Heptacosane	36.86	2717.6	268 m/z	13702
	Hentriacontane	41.22	3125.6	268 m/z, 518	431
	Nonacosane	41.42	3145.4	57 m/z	2007
	unknown_43_26_55: alkene/alkanol	43.22	3333.4	379 m/z	7777
	Tritriacontane	43.26	3337.1	55 m/z	109587
**Alkylresorcinols**	**Bilobol C15:1**	**36.65**	**2698.5**	**180 m/z**	**743253**
	Bilobol C15:0	36.77	2709.0	268 m/z	47258
	Bilobol C17:1, minor isomer 1	37.83	2802.8	319 m/z	6231
	Bilobol C17:2	38.52	2866.3	204 m/z	23532
	Bilobol C17:3	38.83	2894.9	205 m/z	23129
	Bilobol C17:1, minor isomer 2	38.88	2899.5	268 m/z	184819
	Bilobol C17:1	39.00	2910.0	268, 490	57780
	Bilobol C17:0	39.09	2918.8	268 m/z	18883
	unknown: Bilobol 17:1 sugar derivative	39.14	2923.5	268 m/z	131118
	Bilobol C19:3	40.70	3073.5	505 m/z (117)	98785
	Bilobol C19:1	40.87	3091.0	144 m/z	89949
**Terpenes**	unknown_22_46_131: Eremophilene	22.46	1710.8	131 m/z	13284
	Pimaric acid	31.84	2312.3	117 m/z	105856
	Isopimaric acid	31.89	2316.5	73 m/z	34005
	: Farnesol	32.15	2335.5	241 m/z	4739
	: Dehydroabietic acid	32.69	2376.3	143 m/z	5756
	Abietic acid	32.93	2394.8	204 m/z	5856
	unknown_34_45_445: terpenoid	34.36	2507.6	57 m/z (135)	152536
	: Isopimaric acid	35.00	2560.0	266 m/z	12987
	unknown: Lanosterol 1	41.81	3185.1	467 m/z	29901
	unknown: Lanosterol 2	43.10	3319.7	438 m/z (393)	71302
	Lanosterol	43.54	3367.3	438, 422	965240
	: a-amyrin	43.84	3400.0	495 m/z	6659
	unknown: Parkeyl acetate	44.05	3423.3	218 m/z	28071
	: Lupeol	44.40	3461.2	422 m/z 453	677452
	Cycloartenol	44.47	3469.3	190 m/z 498	847408
	Lanosta-8,24-dien-3-ol, acetate, (3β)-	44.59	3482.4	221 m/z (69)	66912
	Olean-12-en-3-ol, acetate, (3β)-	44.73	3499.0	509 m/z	128608
	**Lupeol**	**44.96**	**3524.9**	**218 m/z**	**1215658**

	Lup-20(29)-en-3-ol, acetate, (3β)-	45.36	3570.5	281 m/z (69)	625850
	a Bisabolol	45.42	3576.5	189 m/z (57)	40899
	unknown: Ursolic acid	47.99	3885.3	103 m/z, 495 510	2241
	unknown: Oleanolic acid	48.84	3992.3	197 m/z 287 505	45130
**Sterols**	unknown_34_81_460: sterol	34.74	2538.7	121 m/z, 348 451 466	1111
	unknown_43_10_438: sterol	42.79	3287.6	481 m/z	94507
	unknown_43_22_379: sterol	43.15	3325.0	393 m/z (438)	10753
	**unknown_43_54_438: sterol**	**43.50**	**3362.5**	**57 m/z**	**293338**
	unknown_44_40_422: sterol	44.11	3429.5	177 m/z	141584
	unknown_45_36_281: sterol	45.28	3561.3	189 m/z	9889
	unknown_46_20_496: sterol	45.69	3608.5	523 m/z	60003
	unknown_47_99_510: sterol	47.81	3862.2	551 m/z	2400
	unknown_49_06_117&580: sterol-carboxylate	48.95	4007.0	73 m/z	39534
	unknown_49_77_585: sterol-alkyl derivative	49.06	4020.1	131 m/z 565 580	1227
**Others**	**Phosphoric acid**	**13.11**	**1267.2**	**299 m/z (155)**	**626490**
	: 3-Deoxytetronic acid	16.21	1399.9	103 m/z	9656
	: Terephthalic acid ester	25.87	1909.0	73 m/z	300516
	Uric acid	28.51	2077.9	338 m/z	447
	Guanine	28.73	2092.8	245 m/z (57)	3712
	: Galactosylglycerol	30.80	2236.8	117 m/z	66151
**Unknowns**	unknown_15_20_255	15.20	1355.2	255 m/z	1203
	unknown_34_74_466	34.45	2514.7	237 m/z, 445	3952
	unknown_35_00_266	34.81	2543.7	327 m/z 387, 460	6842
	unknown_35_36_445	35.33	2586.6	121 m/z	9205
	unknown_40_87_144	40.68	3072.1	117 m/z	50726
	**unknown_44_59_221**	**44.54**	**3476.8**	**393 m/z (509)**	**1496782**
	unknown_47_65_524	46.85	3744.6	267, 339, 588	800
	unknown_48_75_143	48.37	3932.8	495 m/z 510 585	5049
	unknown_48_84_505	48.75	3980.1	143 m/z (219)	9624
	unknown_50_63_598	50.63	4228.7	121 m/z 490 598	1042
