# Supplementary material for: Chemical Profiling and Geographic Differentiation of Ugandan Propolis by GC-MS Through Chemometric Modelling
Source: Molecules. 2025 Nov 16;30(22):4435. doi: 10.3390/molecules30224435 (PMC12655498; doi:10.3390/molecules30224435)
Supplement: Supplementary file 1 [file molecules-30-04435-s001.zip › Supplementary file S3.pdf]

Table S1A oPLS-DA Analysis of Volatile Compounds in Propolis Samples

**T Score (%)**	**Orth. Score (%)**	**R^2Y**	**Q^2**	**Permutations (n = 20), p < 0.05**	**Pairwise Comparison**
24.0	18.6	0.933	0.879	Q2: 0.974, R^2Y: 0.996	ADJ-KIB
36.6	14.2	0.939	0.923	Q2: 0.978, R^2Y: 0.992	ADJ-MBA
48.3	13.5	0.953	0.932	Q2: 0.985, R^2Y: 0.994	MAS-BUS
19.5	19.9	0.919	0.844	Q2: 0.954, R^2Y: 0.991	ADJ-NAK
20.9	14.3	0.973	0.910	Q2: 0.963, R^2Y: 0.994	LIR-NAK
41.3	17.1	0.927	0.911	Q2: 0.988, R^2Y: 0.996	ADJ-RWA
35.6	16.8	0.900	0.872	Q2: 0.970, R^2Y: 0.993	KIB-MBA
55.8	11.8	0.968	0.950	Q2: 0.989, R^2Y: 0.997	KIB-BUS
45.2	22	0.884	0.862	Q2: 0.982, R^2Y: 0.992	KIB-RWA
40.5	21.3	0.909	0.887	Q2: 0.990, R^2Y: 0.995	KOT-RWA
28.9	19.5	0.972	0.938	Q2: 0.979, R^2Y: 0.993	KOT-MAS
38.6	22.4	0.870	0.834	Q2: 0.975, R^2Y: 0.989	MAS-RWA
29.4	12.5	0.960	0.924	Q2: 0.973, R^2Y: 0.993	KOT-NAK
28.4	19.2	0.903	0.854	Q2: 0.965, R^2Y: 0.988	LIR-MAS
25.0	20.1	0.882	0.812	Q2: 0.947, R^2Y: 0.984	MAS-MBA
35.7	14.4	0.930	0.909	Q2: 0.979, R^2Y: 0.992	LIR-MBA
43.7	16.5	0.914	0.894	Q2: 0.985, R^2Y: 0.993	LIR-RWA
32.6	15.1	0.949	0.926	Q2: 0.983, R^2Y: 0.994	KOT-ADJ
50.7	17.3	0.964	0.944	Q2: 0.985, R^2Y: 0.993	KOT-BUS
30.8	13.1	0.973	0.948	Q2: 0.987, R^2Y: 0.996	KOT-KIB
21.3	22.4	0.908	0.836	Q2: 0.982, R^2Y: 0.996	KOT-LIR
32.8	16.9	0.960	0.933	Q2: 0.983, R^2Y: 0.995	KOT-MBA
23.9	16.0	0.931	0.878	Q2: 0.977, R^2Y: 0.993	LIR-ADJ
50.8	12.5	0.968	0.952	Q2: 0.990, R^2Y: 0.997	LIR-BUS
40.9	20.3	0.925	0.901	Q2: 0.984, R^2Y: 0.994	NAK-RWA
21.8	19.4	0.946	0.888	Q2: 0.961, R^2Y: 0.991	LIR-KIB
20.4	15.0	0.886	0.790	Q2: 0.928, R^2Y: 0.986	MAS-NAK
20.9	18.3	0.888	0.792	Q2: 0.941, R^2Y: 0.996	MAS-KIB
35.4	24.6	0.820	0.768	Q2: 0.963, R^2Y: 0.991	MBA-BUS
23.8	33.0	0.633	0.561	Q2: 0.979, R^2Y: 0.995	MBA-RWA
32.9	15.1	0.947	0.919	Q2: 0.964, R^2Y: 0.989	NAK-MBA
50.0	10.9	0.980	0.966	Q2: 0.992, R^2Y: 0.997	NAK-BUS
16.7	14.7	0.978	0.859	Q2: 0.927, R^2Y: 0.991	NAK-KIB
20.2	45.5	0.812	0.680	Q2: 0.972, R^2Y: 0.994	RWA-BUS
28.2	18.8	0.862	0.826	Q2: 0.968, R^2Y: 0.994	ADJ-MAS

Table S2B oPLS-DA Analysis of Chemical Compounds in Derivatised Propolis Samples

	OPLSDA parameters				
**T Score (%)**	**Orthogonal Score (%)**	**R^2Y**	**Q^2**	**Permutations (n = 20)**, $p < 0.05$	**Pairwise Comparison**
25.0	15.9	0.961	0.907	Q2: 0.921, R^2Y: 0.980	ADJ-MAS
16.4	14.3	0.906	0.787	Q2: 0.895 , R^2Y: 0.968	LIR-MAS
15.8	18.7	0.945	0.794	Q2: 0.890 , R^2Y: 0.995	ADJ-KIB
23.6	16.9	0.969	0.900	Q2: 0.942 , R^2Y: 0.988	KOT-KIB
16.2	14.1	0.880	0.725	Q2: 0.876 , R^2Y: 0.984	RWA-BUS
20.7	16.4	0.971	0.884	Q2: 0.933 , R^2Y: 0.990	ADJ-RWA
22.5	13.1	0.884	0.821	Q2: 0.894 , R^2Y: 0.972	MAS-KIB
15.2	10.5	0.967	0.816	Q2: 0.907 , R^2Y: 0.991	MAS-RWA
14.5	22.5	0.891	0.728	Q2: 0.911 , R^2Y: 0.995	KIB-LIR
26.0	18.1	0.855	0.791	Q2: 0.948 , R^2Y: 0.998	NAK-KIB
28.1	12.4	0.957	0.918	Q2: 0.960 , R^2Y: 0.990	KIB-RWA
19.2	16.7	0.947	0.842	Q2: 0.923 , R^2Y: 0.990	KOT-ADJ
14.8	16.5	0.939	0.752	Q2: 0.865 , R^2Y: 0.973	LIR-ADJ
21.0	17.6	0.891	0.788	Q2: 0.924 , R^2Y: 0.998	ADJ-NAK
26.8	15.7	0.977	0.933	Q2: 0.973 , R^2Y: 0.997	ADJ-BUS
17.6	19.1	0.948	0.864	Q2: 0.933 , R^2Y: 0.993	ADJ-MBA
24.2	13.4	0.989	0.943	Q2: 0.965 , R^2Y: 0.995	MAS-BUS
17.0	14.8	0.945	0.842	Q2: 0.927 , R^2Y: 0.988	KOT-MAS
18.5	21.0	0.793	0.703	Q2: 0.924 , R^2Y: 0.993	MAS-MBA
12.0	21.6	0.880	0.633	Q2: 0.847 , R^2Y: 0.993	MAS-NAK
24.4	22.1	0.934	0.893	Q2: 0.969 , R^2Y: 0.994	KIB-MBA
13.4	20.7	0.905	0.746	Q2: 0.878 , R^2Y: 0.988	KOT-MBA
16.4	13.6	0.964	0.847	Q2: 0.942 , R^2Y: 0.990	KOT-RWA
21.0	15.3	0.948	0.890	Q2: 0.947 , R^2Y: 0.988	KOT-BUS
17.2	20.1	0.930	0.842	Q2: 0.922 , R^2Y: 0.993	MBA-BUS
13.3	22.8	0.802	0.657	Q2: 0.904 , R^2Y: 0.996	MBA-RWA
